# The Cpn10(1) Co-Chaperonin of *A. thaliana* Functions Only as a Hetero-Oligomer with Cpn20

**DOI:** 10.1371/journal.pone.0113835

**Published:** 2014-11-24

**Authors:** Anna Vitlin Gruber, Gal Zizelski, Abdussalam Azem, Celeste Weiss

**Affiliations:** Department of Biochemistry and Molecular Biology, Tel Aviv University, Tel Aviv, Israel; Hokkaido University, Japan

## Abstract

The *A. thaliana* genome encodes five co-chaperonin homologs, three of which are destined to the chloroplast. Two of the proteins, Cpn10(2) and Cpn20, form functional homo-oligomers *in vitro*. In the current work, we present data on the structure and function of the third *A. thaliana* co-chaperonin, which exhibits unique properties. We found that purified recombinant Cpn10(1) forms inactive dimers in solution, in contrast to the active heptamers that are formed by canonical Cpn10s. Additionally, our data demonstrate that Cpn10(1) is capable of assembling into active hetero-oligomers together with Cpn20. This finding was reinforced by the formation of active co-chaperonin species upon mixing an inactive Cpn20 mutant with the inactive Cpn10(1). The present study constitutes the first report of a higher plant Cpn10 subunit that is able to function only upon formation of hetero-oligomers with other co-chaperonins.

## Introduction

Chaperonins are a family of ∼1000 kDa cylindrical protein complexes, composed of two partners, the 60 kDa chaperonin (Cpn60) and the 10 kDa co-chaperonin (Cpn10). Together, these chaperones provide a compartment in which a substrate polypeptide is isolated from the cellular environment and can safely fold in an ATP - dependent manner [1]. The prototypical system found in *E. coli* provides the bulk of our knowledge on the oligomeric chaperonin complexes. For this system, high resolution structures of the GroEL tetradecamer (Cpn60) and its co-chaperonin, the GroES heptamer (Cpn10), have been solved by X-ray crystallography both individually and in a variety of complexes [2]–[7]. Two seven-membered rings of GroEL form the main chaperonin structure in which the substrate protein finds protection. Seven subunits of GroES assemble into dome-like structures, which cap the ends of the GroEL cylinder and induce formation of the protein-folding chamber. The interaction between the two oligomers is mediated by a ∼20 residue sequence of GroES, known as the ‘mobile loop’, an unstructured region that adopts a β-hairpin conformation when bound to GroEL [2], [8], [9]. At the other end of the GroES subunit is an additional loop, seven of which form a roof-like ceiling in the heptameric co-chaperonin oligomer.

In contrast to the simple homo-oligomeric structures found in *E. coli*, chloroplast chaperonins are unique in three aspects: 1) Chloroplasts harbor duplicated chaperonin and co-chaperonin genes. For example, chloroplasts generally contain two chaperonin subtypes, Cpn60α and Cpn60β. They also harbor two structurally distinct co-chaperonin proteins, the gene of one (cpn10) which encodes for a 10 kDa subunit, and the gene of the second (cpn20), which consists of two homologous cpn10 sequences joined head-to-tail. 2) Often, greater than one gene for each subtype is present in the genome. 3) Both chaperonin and co-chaperonin subunits can assemble into a variety of functional homo- and hetero-oligomeric complexes. Cpn60αβ hetero-oligomer is the most abundant oligomeric form *in vivo* (reviewed in [10]), although the Cpn60β subunits were shown to be functional as homo-oligomers *in vitro* [11]. In terms of co-chaperonins, Cpn10 and Cpn20 can function *in vitro* as heptameric and tetrameric homo-oligomers, respectively [12]–[15]. In addition, hetero-oligomers of Cpn20 and Cpn10 were shown to form and be functional *in vitro* [16].

*A. thaliana* contains three co-chaperonin genes with predicted chloroplast transit peptides: Cpn20 (At5g20720), Cpn10(1) (At3g60210) and Cpn10(2) (At2g44650), the latter two of which exhibit 84% sequence identity. Cpn10(1) was not detected in proteomics studies, although, mRNA analyses demonstrate that this gene is expressed at low levels [17]. In contrast, Cpn20 and Cpn10(2) were both detected at the mRNA level, identified in proteomic studies [17], [18] and characterized *in vitro* [12]–[14], [19], [20].

In the present work, we show that, despite the high similarity to Cpn10(2) at the primary sequence level, Cpn10(1) exhibits unique structural properties and is functional only when it is integrated into a hetero-oligomeric complex with Cpn20. Mixtures of Cpn10 and Cpn20 contain hetero-oligomeric species that co-exist together with Cpn20 homo-oligomers, increasing complexity and flexibility of the chloroplast chaperonin system.

## Materials and Methods

### Purification of recombinant co-chaperonin proteins

Cpn10(1) lacking the N-terminal chloroplast transit peptide was cloned into the pET22b^+^ vector between XhoΙ and NdeΙ restriction enzymes and transformed into the *E. coli* BL21(T7^+^). Serine 43 was chosen as the first amino acid, based on alignment with Cpn10(2) [12].

Cpn10(1) was purified based on the Cpn10(2) purification protocol [12] with several differences. Briefly, *E. coli* BL21 (T7^+^) strain, carrying the pET22b^+^ - Cpn10(1) plasmid, was grown overnight at 37°C in LB medium containing 200 µg/ml ampicillin and 34 µg/ml chloramphenicol. Next, the starter was diluted 1∶30 and grown at 37°C to an OD_600_ of 0.6. The induction of Cpn10(1) expression was initiated by the addition of 1 mM IPTG and the bacteria was incubated in a shaker at 16°C overnight. Cells were harvested, resuspended in lysis buffer (1∶10 w/v) containing: 100 mM Tris-HCl pH 8, 10 mM EDTA, 1 mM DTT, 0.1 mg/ml DNase I (Sigma-Aldrich) and 0.5 mM PMSF (Roche) and disrupted by microfluidizer. The lysate was spun at 14000 rpm for 1 hour at 4°C and the supernatant was filtered through a 0.2 µM filter (Whatman) and loaded onto an anion exchange column (Mono-Q, GH Healthcare), that was pre-washed with buffer D (20 mM Na-HEPES pH 7.4 and 5% (v/v) glycerol). Bound proteins were eluted from the column with a linear gradient (0%–50%, 90 ml and flow rate of 1 ml/min) of buffer E (20 mM Na-HEPES pH 7.4, 1M NaCl and 5% (v/v) glycerol). Enriched fractions were pooled and concentrated to 3 ml, which was loaded onto a Superdex 200 gel filtration column pre-equilibrated with buffer F (20 mM Na-HEPES pH 7.4, 100 mM NaCl, 5 mM MgCl_2_). The column was developed at 1 ml/min (4°C). Relevant fractions were collected, concentrated to 3 ml and loaded onto a Superdex 75 gel filtration column (GE healthcare) equilibrated with buffer F. Relevant fractions were collected, concentrated and the protein concentration was determined using the Bicinchoninic Acid Protein Assay kit (Sigma), with BSA as a standard. All molar protein concentrations refer to protomer concentration unless otherwise indicated.

Previously published protocols were used to purify GroES, Cpn20, G32A, Cpn10(2) [12] and mouse mt-cpn10 [21].

### Purification of recombinant chaperonin proteins

Previously published protocols were used to purify Cpn60β3 (At1g55490) [11] and GroEL [20].

Cpn60α2 (At2g28000) cloning and purification was based on the Cpn60β2 method [11] with the following adaptation: Cpn60α2 was cloned between the BamHI-NotI sites of the modified version of pET21d+ which codes for an octa-histidine tag followed by the TEV (Tobacco Etch virus) proteolysis site at the amino terminus of the protein [22]. The first amino acid of the mature protein was chosen to be alanine 46 and it contained an additional glycine-serine at the N-terminus. The construct was expressed in *E. coli* Rosetta (Novagen) and purified as described in [11].

### Reconstitution of Cpn60αβ hetero-oligomers from individual monomers

Previous studies have reported the reconstitution of active chloroplast chaperonin homo- or hetero-oligomers from monomers [11], [23]. The same protocol is used in this study with minor modifications as follows: The reconstitution mixture containing 50 mM Tris-HCl pH 8, 300 mM NaCl, 10 mM MgCl_2_, 16 mM KCl, 2 mM dithiothreitol (DTT), 5 mM ATP, 150 µM Cpn60α2, 100 µM Cpn60β3 and 50 µM mt-cpn10, was incubated for 5 minutes at room temperature and then for 1 hour at 30°C. For oligomer purification, oligomers and monomers in the reconstitution reaction were separated using a Superdex 200 gel filtration column pre-equilibrated with buffer containing 50 mM Tris-HCl pH 8, 300 mM NaCl and 5% glycerol at room temperature. Fractions containing oligomers were pooled, and treated with Ni–NTA-agarose beads in order to remove any traces of his-tagged mt-cpn10 that might have co-purified with the Cpn60. The relevant fractions were concentrated and flash frozen in liquid nitrogen.

### *In vitro* refolding of urea-denatured MDH

Malate dehydrogenase (35 µM MDH - Roche) was denatured in 4.5 M urea and 5 mM DTT for 3 hours at room temperature. The refolding reaction was carried out in two steps. First, MDH was rapidly diluted to 0.5 µM into a refolding solution containing 10 µM GroEL (or chloroplast Cpn60αβ) in 50 mM Tris-HCl pH 7.4, 10 mM MgCl_2_, 50 mM KCl, 5 mM DTT and incubated for 15 min at 37°C to form the chaperonin-MDH binary complex. Next, the refolding of MDH was initiated by adding different concentrations of co-chaperonins or co-chaperonin mixtures (as stated in Figures) and 5 mM ATP. After a 30 min incubation at 37°C, 10 µl aliquots were assayed for MDH activity by adding 990 µl of reaction mixture containing 150 mM K-phosphate pH 7.6, 10 mM DTT, 0.28 mM NADH and 0.5 mM oxaloacetate and monitoring the oxidation of NADH at 340 nm.

### Cross-linking

45 µM co-chaperonin was cross-linked with 1 mM disuccinimidyl suberate (DSS - Pierce) at room temperature in 50 mM Na-HEPES pH 7.5, 10 mM MgCl_2_ and 100 mM KCl. The cross-linking reaction was stopped by the addition of a one-third volume of sample buffer: 62.5 mM Tris-HCl pH 6.8, 2% SDS, 5% β-mercaptoethanol, 20% glycerol, 1 M urea and boiling for 5 min, prior to denaturing electrophoresis in a mini 10–19% acrylamide gradient gel.

### Reconstitution of β1 homo-oligomers

The reconstitution reaction was carried out in 50 mM Tris-HCl pH 8, 0.3 M NaCl, 10 mM MgCl_2_, 16 mM KCl, 2 mM dithiothreitol (DTT), 5 mM ATP, 50 µM Cpn60β1 monomer and 25 µM of different co-chaperonins or co-chaperonin mixtures. The reconstitution mixture was incubated for 5 minutes at room temperature and then for 1 hour at 30°C. 3 µl of the reconstitution reaction was loaded on a 6% native polyacrylamide gel.

## Results

### The oligomeric structure of recombinant Cpn10(1) in solution

**Figure 1:**
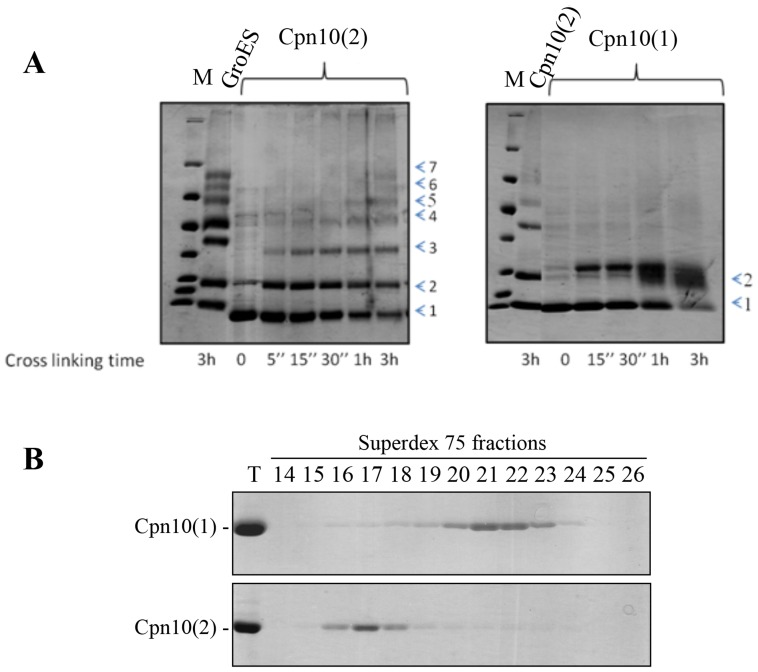
The oligomeric state of Cpn10(1). (A) Time-dependent crosslinking pattern of Cpn10(1) (right panel) and Cpn10(2) (left panel). 45 µM of co-chaperonin was exposed to 1 mM DSS for the indicated times. The crosslinking products were separated on a mini gradient SDS acrylamide gels (10–19%), followed by staining with Coomassie blue. The numbers at the right indicate the number of cross-linked subunits in each species. (B) Elution profile of ∼1 mg Cpn10(1) (top panel) or ∼1 mg Cpn10(2) (lower panel) separated by gel filtration on a Superdex 75 column at room temperature at a flow rate of 1 mg/ml. Fractions were analyzed by SDS-PAGE (10 µl per lane). T = 5 µg of Cpn10. Fractions 15–18 contain primarily heptamer while fractions 20–23 contain primarily monomer - dimer.

The *Arabidopsis* Cpn20 and Cpn10(2) have been characterized structurally and functionally by our group and others in the past [12]–[14], [19], [20]. The third co-chaperonin homolog - Cpn10(1) - has not yet been studied *in vitro* or *in vivo* and no obvious phenotype was found for a knockout strain [24]. The active form of most known single-domained co-chaperonins (for example: GroES, mt-cpn10 and Cpn10(2)) is a heptamer. Thus, we wondered whether Cpn10(1) has a similar oligomeric state *in vitro*. In order to answer this question, we purified the recombinant Cpn10(1) and carried out cross-linking with DSS. As can be seen in Figure 1A, Cpn10(2) and GroES exhibit a cross-linking pattern that is typical for heptameric oligomers, as indicated by the time-dependent accumulation of seven bands of cross-linking products [25]. Surprisingly, the cross-linking of Cpn10(1) yielded only two, low molecular-weight cross-linking products. These results suggest that Cpn10(1) retains a unique oligomeric state in solution and exists in dimeric form or as a mixture of dimers and monomers. An elution profile from the Superdex 75 gel filtration column supported these results, since the majority of the Cpn10(1) protein eluted in fractions 20–23, in contrast to Cpn10(2), which eluted primarily in fractions 16–18 (Figure 1B).

### Cpn10(1) is active only upon formation of hetero-oligomers with Cpn20

**Figure 2:**
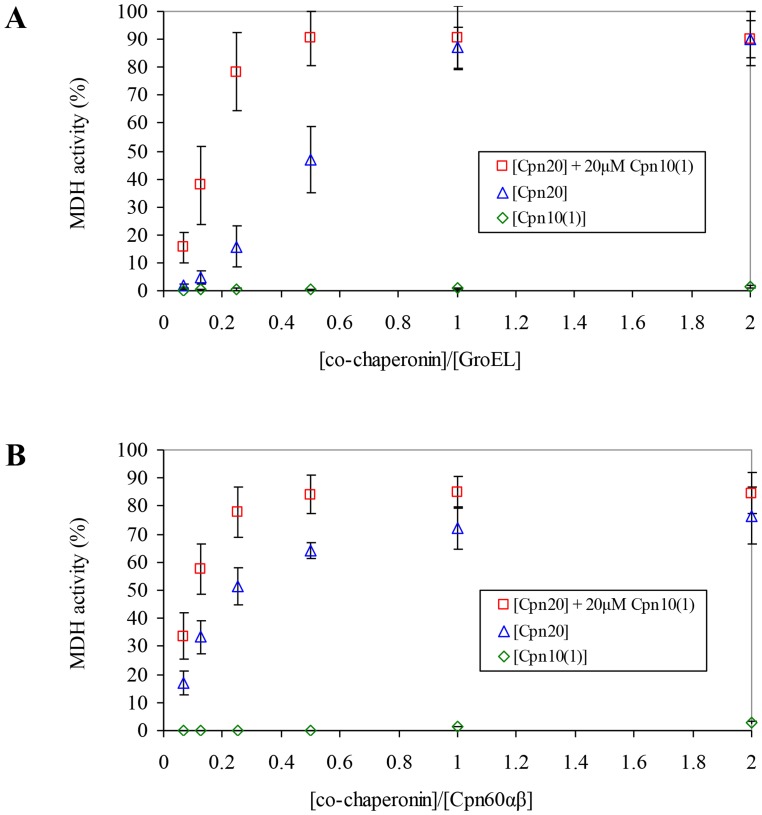
Effect of Cpn10(1) on co-chaperonin activity of Cpn20. MDH refolding was carried out by either (A) GroEL or (B) Cpn60αβ, as described in Materials and Methods, in the presence of increasing concentrations of various co-chaperonin species, as indicated. 100% was taken as the activity of GroEL assisted by a saturating concentration of GroES, or the activity of Cpn60αβ assisted by mt-cpn10. Results are presented as an average of 3 independent experiments ± SD.

Given the unusual oligomeric structure of Cpn10(1), it was pertinent to examine whether this dimeric co-chaperonin was able to assist chaperonin in mediating the refolding of denatured protein. As can be seen in Figure 2, Cpn20 was functional at assisting both GroEL and chloroplast chaperonin in refolding urea-denatured MDH. In contrast, Cpn10(1) was not able to assist chaperonins in mediating protein folding. This result was surprising, given its high homology to Cpn10(2), which was fully functional with GroEL [12].

The fact that purified Cpn10(1) is not active in assisting protein folding raises the question as to why plants harbor a non-functional species? The answer to this question may be provided by a recent study showing that *C. reinhardtii* harbors three co-chaperonin forms, which are functional only upon incorporation into mixed hetero-oligomeric co-chaperonin species. The authors demonstrated that the *A. thaliana* Cpn10(2) is also capable of forming functional mixed hetero-oligomers with the *Arabidopsis* Cpn20 *in vitro* [16]. Since both co-chaperonins, Cpn20 and Cpn10(2), were active individually as homo-oligomers [12], [13], [19], the functional significance of the formed hetero-oligomers remained obscure.

In light of the above research, we examined the possibility that Cpn10(1) acquires functionality by assembling with the other *A. thaliana* chloroplast co-chaperonins. For this purpose, we carried out refolding experiments using GroEL or Cpn60αβ (10 µM), which was supplemented with a constant concentration of inactive Cpn10(1) (20 µM) and increasing concentrations of Cpn20 (0.625–20 µM). Since Cpn10(1) alone was not able to assist chaperonin in mediating protein folding at any concentration, we assumed that any increase in the refolding yield above that of Cpn20 alone, would be due to the formation of mixed hetero-oligomers.

Interestingly, a clear synergistic effect can be seen in Figure 2 upon titrating Cpn20 in the presence of a constant concentration of Cpn10(1). At low concentrations of Cpn20 (for example 0.25 ratio of [co-chaperonin]/[GroEL]), where the refolding yield is sub-saturating for Cpn20 alone, the presence of Cpn10(1) resulted in a 5-fold increase in refolding activity (15.71% and 78.26% respectively - Figure 2A). A similar effect was seen using Cpn60αβ (Figure 2B). In this case, the increase in refolding was from 51.39% to 77.83%. These results suggest that Cpn20 and Cpn10(1) combined to form functional hetero-oligomeric species.

**Figure 3:**
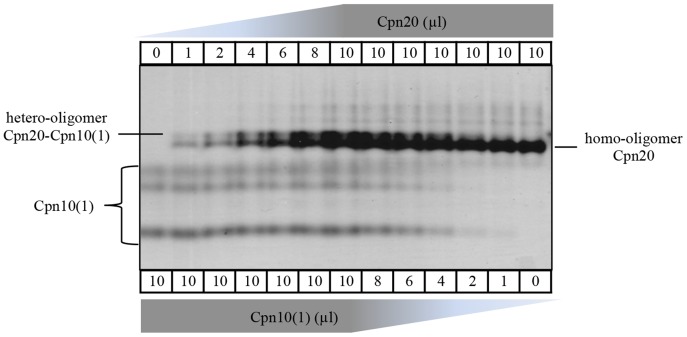
Visualization of Cpn10(1)-Cpn20 hetero-oligomer by native gel. Co-chaperonin proteins were diluted to 100 µM each (protomer) and then mixed to obtain the indicated ratios. After five minutes at room temperature, native sample buffer was added to a 50 µl final volume and 10 µl of the mixture was loaded on a 6% native acrylamide gel. The identity of the different co-chaperonin species is indicated to the sides of the gel. An additional band above that of Cpn20 can be clearly seen in samples that also contain Cpn10(1). Note that in the native gels, Cpn10(1) appears as three different species.

In order to further explore the hetero-oligomerization, we separated mixtures containing different Cpn20/Cpn10(1) ratios on a native gel. As can be seen in Figure 3, upon mixing these two co-chaperonins, a new band appears above Cpn20, representing the formation of a Cpn20-Cpn10(1) hetero-oligomer. It can be seen that Cpn20 homo-oligomer never dissociates completely, even in the presence of a 10-fold molar excess of Cpn10(1). In this regard, it is also interesting to note that hetero-oligomer formation is spontaneous and does not depend on any other factors like Cpn60 or ATP.

**Figure 4:**
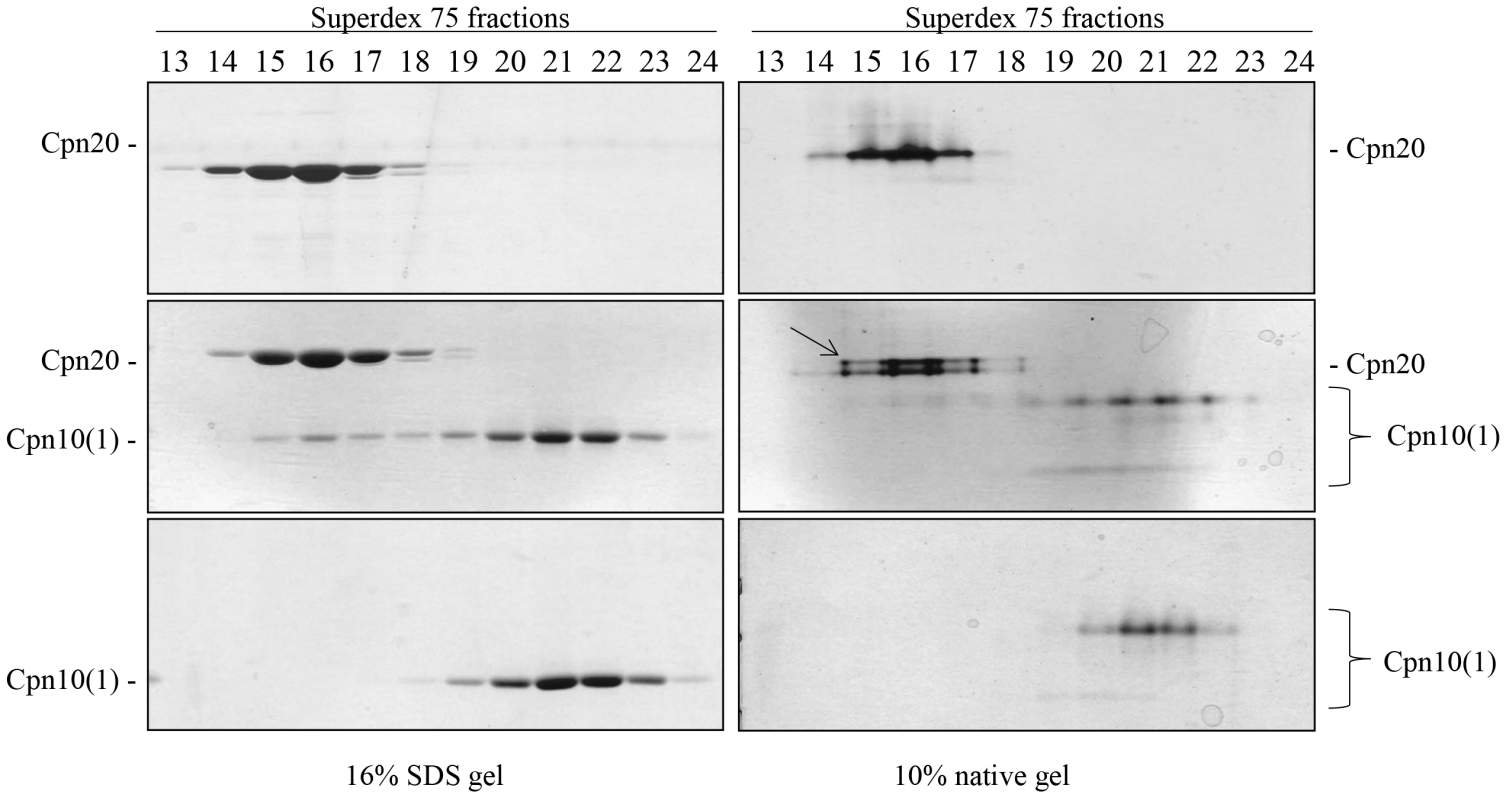
Analysis of hetero-oligomer formation by gel filtration. Elution profile of ∼1.5 mg Cpn20 (top panels), ∼1.5 mg Cpn10(1) (bottom panels) or a mixture of the two (middle panels), separated by gel filtration on a Superdex 75 column at 4°C at a flow rate of 1 mg/ml. Fractions were subsequently analyzed by either SDS (left panel) or native (right panel) PAGE (20 µl per lane). Cpn20 and Cpn10(1) are indicated for each gel. The hetero-oligomeric species is marked with an arrow on the native gel. Fractions 14–17 contain primarily 70–80 kDa species while fractions 20–23 contain primarily 10–20 kDa species.

To obtain additional direct evidence of hetero-oligomer formation, we analyzed mixtures of Cpn20 and Cpn10 by gel filtration. Fractions eluting from the column were separated by both native and denaturing electrophoresis. In Figure 4, left panel, it can be seen that Cpn10(1) by itself elutes from the column in fractions 19–24, while Cpn20 elutes in fractions 14–18. Upon mixing the two co-chaperonins, a shift was seen in the elution profile of Cpn10(1), with additional Cpn10(1) observed in fractions 15–18, the area of Cpn20 elution. The same fractions were separated on native acrylamide gels (Figure 4, right panel). Consistent with the formation of hetero-oligomer, elution of the Cpn10(1)-Cpn20 mixture yielded an additional band (marked by an arrow) above the Cpn20 band, in fractions 15–18. These results confirm the formation of a novel species containing both subunit types.

### Cpn10(1) forms functional hetero-oligomeric species with an inactive Cpn20 mutant

**Figure 5:**
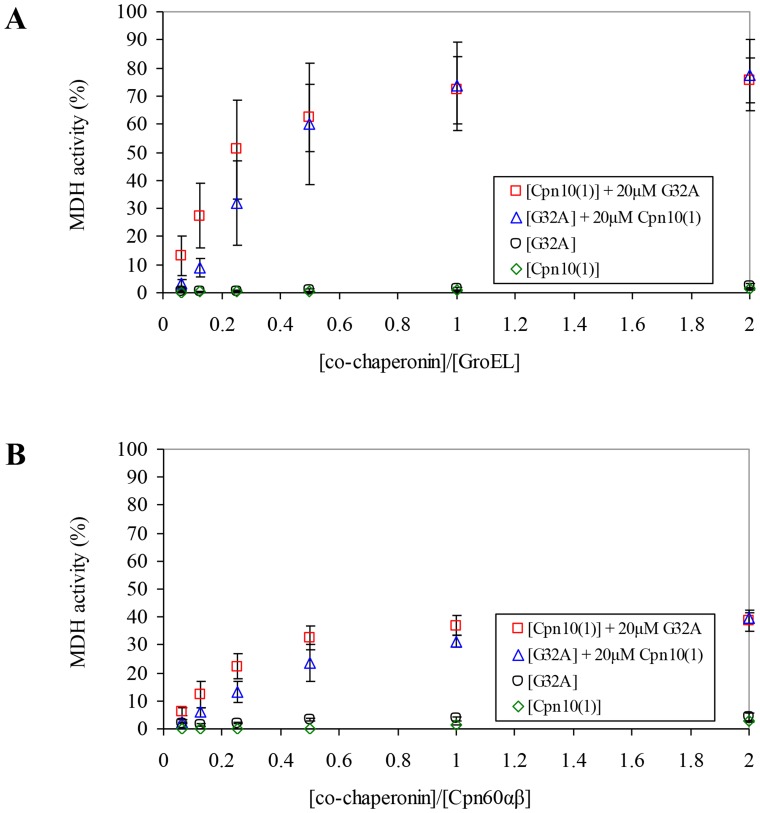
Functional synergism between inactive co-chaperonin species Cpn10(1) and G32A. MDH refolding was carried out by either (A) GroEL or (B) Cpn60αβ, as described in Materials and Methods, in the presence of increasing concentrations of various inactive co-chaperonin species, as indicated. 100% was taken as the activity of GroEL assisted by a saturating concentration of GroES, or the activity of Cpn60αβ assisted by mt-cpn10. Results are presented as an average of 3 independent experiments ± SD.

To further support our results, we used a Cpn20 mutant, G32A, which was previously characterized in our laboratory [20]. This Cpn20 mutant is modified at a highly conserved glycine, which was shown to be important for the flexibility of the mobile loop – the region that facilitates binding of co-chaperonin to Cpn60. G32 is located in the N-domain of Cpn20 and its substitution to alanine results in a co-chaperonin which is inactive at 37°C. As can be seen in Figure 5, while both G32A and Cpn10(1) are individually inactive, their combination resulted in ∼75% co-chaperonin activity with GroEL (Figure 5A) and ∼40% using chloroplast Cpn60αβ (Figure 5B). Thus the presence of small amount of G32A can serve as a template for Cpn10(1) oligomerization, resulting in active mixed oligomers. Reciprocally, the presence of Cpn10(1) molecule in the mixed oligomer is sufficient to fully rescue co-chaperonin activity of the inactive G32A mutant. While the general refolding trends using chloroplast Cpn60αβ were similar to those using GroEL, some differences were observed in the refolding pattern or yields. For example, Cpn60αβ refolding yields are much lower with G32A and Cpn10(1), than they are using GroEL. This might be due to a greater sensitivity of Cpn60αβ to mutations in the endogenous co-chaperonin partner.

**Figure 6:**
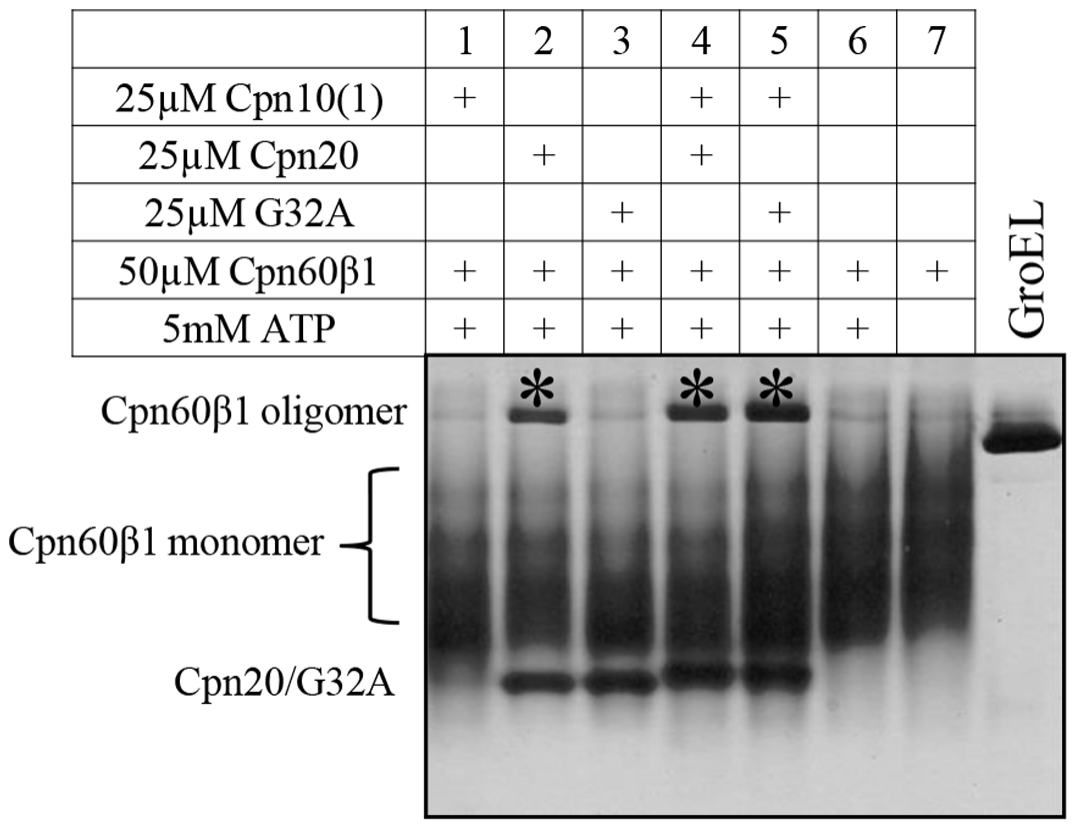
Effect of various co-chaperonins on the reconstitution of Cpn60β1 oligomers. Cpn60β1 oligomer was assembled from monomeric form as described in Materials and Methods, in the presence of Mg-ATP and various co-chaperonins or co-chaperonin mixtures as indicated. 3 µl of reconstitution reaction was loaded on a 6% native polyacrylamide gel. The reconstituted oligomers are indicated by an asterisk. GroEL oligomer is presented as a control.

An additional method was used in order to demonstrate the functional interaction between Cpn10(1) and Cpn20. We took advantage of the ability of various co-chaperonins to induce the oligomerization of Cpn60β monomers, as demonstrated in Vitlin et al., [11]. Consistent with their inability to assist Cpn60-facilitated refolding, neither Cpn10(1) nor G32A alone was able to stimulate the formation of Cpn60β1 oligomers (Figure 6, lane 1 and 3 respectively). In contrast, a mixture of the two inactive co-chaperonins had an effect that was similar to that observed when wild type Cpn20 was added (Figure 6, lane 5 and 2 respectively), resulting in formation of Cpn60β1 oligomer.

**Figure 7:**
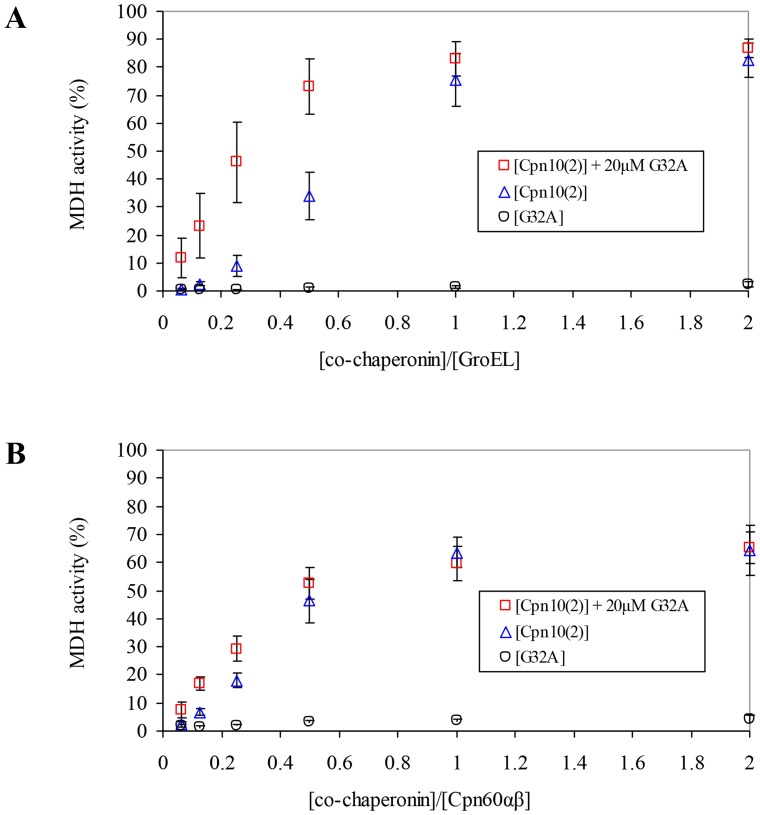
Effect of G32A on co-chaperonin activity of Cpn10(2). MDH refolding by either A) GroEL or B) Cpn60αβ was carried out as described in Materials and Methods in the presence of increasing concentrations of various co-chaperonin species, as indicated. 100% was taken as the activity of GroEL assisted by a saturating concentration of GroES, or the activity of Cpn60αβ assisted by mt-cpn10. Results are presented as an average of 3 independent experiments ± SD.

The inactive mutant G32A was also useful as a tool allowing us to examine hetero-oligomers formed by Cpn10(2) and Cpn20. We found that titration of Cpn10(2) on a background of 20 µM G32A resulted in a significant increase in GroEL activity (Figure 7A). For example, at a 0.25 ratio of [co-chaperonin]/[GroEL]), where the refolding yield is sub-saturating for Cpn10(2) alone, the addition of G32A resulted in an approximately 5-fold increase in refolding activity (8.95% versus 46.16% respectively). Addition of G32A to Cpn10(2) also resulted in greater refolding yields using Cpn60αβ, however, the increase was less apparent. At a 0.25 ratio of [co-chaperonin]/[Cpn60αβ], Cpn10(2) gave a refolding yield of 18.06%, vs 29.15% in the presence of G32A (Figure 7B). These results indicate that functional hetero-oligomers are formed between Cpn20 and Cpn10(2), as previously demonstrated [16]. Similar experiments titrating Cpn10(2) on a background of 20 µM Cpn10(1) did not yield an increase in activity over that of Cpn10(2) alone (not shown), suggesting that the two Cpn10s may not assemble to form hetero-oligomeric species with each other.

## Discussion

The chloroplast co-chaperonin system continues to challenge us with structural and functional divergence from the *E.coli* system. One major difference is the existence of a double co-chaperonin gene (cpn20) whose protein product seems to form tetrameric complexes. The second is the existence of multiple co-chaperonin homologs in plastids. In some organisms the cpn10 gene is duplicated, in some cpn20 and in some, there are multiple copies of both [17], [26], [27]. A third difference, only recently discovered, is the ability of distinct co-chaperonin species to form functional hetero-oligomers [16]. *C. reinhardtii* harbors three expressed co-chaperonin subunits in its chloroplast, none of which is active on its own. Upon mixing, a range of different hetero-oligomeric species can be formed. However, only combinations incorporating the CrCpn10 subunit exhibit any co-chaperonin activity [16]. Thus, it seems that in *C. reinhardtii* system the hetero-oligomer is obligatory for function.

In contrast to the *C. reinhardtii* system, in *A. thaliana*, both Cpn10(2) and Cpn20 can form fully functional homo-oligomers [12], [13], [19]. In the present work, we found that the third co-chaperonin from *A. thaliana*, Cpn10(1), forms inactive, low molecular weight species in solution. Our results additionally showed that subunits of both Cpn10(1) and Cpn10(2) can combine with Cpn20 to form active hetero-oligomers. This was shown by: i) The increase in activity resulting from addition of inactive Cpn10(1) to Cpn20 (Figure 2), ii) The hetero-oligomeric species observed by native gels and gel filtration (Figure 3 and 4), iii) The ability to reconstitute functional, protein folding co-chaperonin by combining inactive Cpn10(1) and an inactive Cpn20 mutant (G32A) (Figure 5), iv) The ability of G32A–Cpn10(1) hetero-oligomers to stimulate formation of Cpn60β1 oligomers (Figure 6) and v) The ability of inactive G32A to enhance the activity of Cpn10(2) (Figure 7).

One of the questions that arises from our data concerns the gain of activity exhibited by G32A (the Cpn20 mutant bearing a mutation in the N-domain mobile loop) at 37°C when supplemented with a very small concentration of Cpn10(1), which is itself inactive (Figure 5). It was previously demonstrated that at least four functional GroES subunits out of seven are necessary for binding GroEL [28]. Assuming one C-terminal domain of the Cpn20 molecule is excluded from the ring as previously suggested [16], this leaves only three functional C-domain subunits out of seven domains, which could explain the non-functionality of the tetrameric G32A oligomer. Upon addition of Cpn10(1), the number of intact subunits rises to four, which could allow the binding to the chaperonin and result in a functional complex.

**Figure 8:**
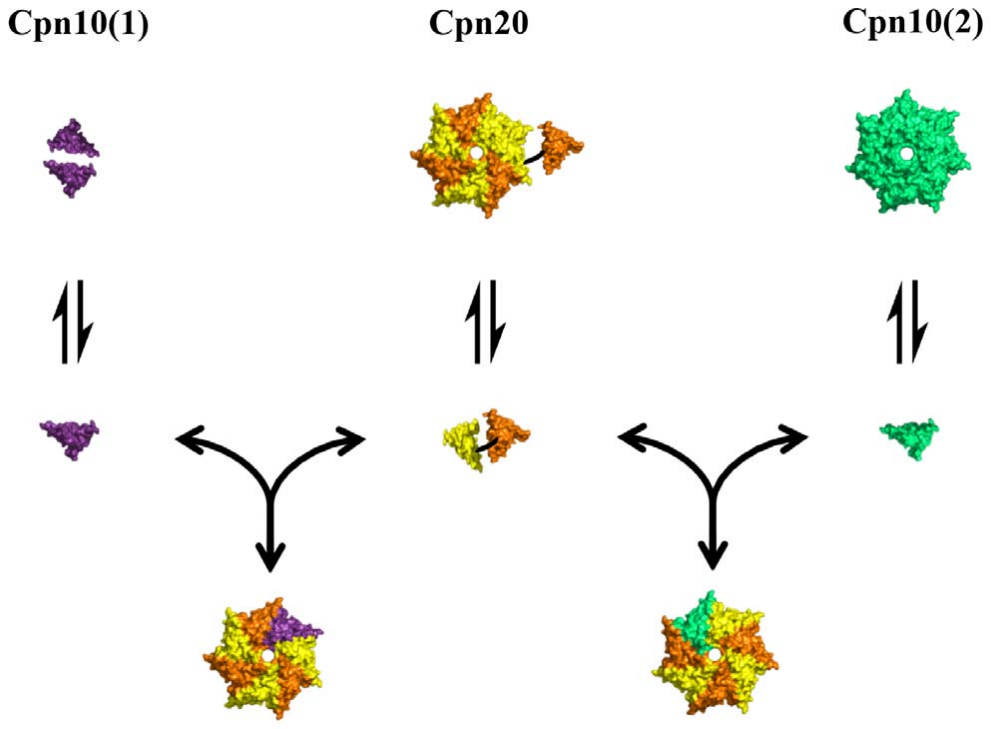
Schematic depiction of equilibria between various co-chaperonin species of *A. thaliana*. Key: Cpn10(1) subunit - purple, Cpn10(2) subunit - green, Cpn20 subunit - orange (N-terminus) and yellow (C-terminus).

How are Cpn10 and Cpn20 molecules arranged in the co-chaperonin hetero-oligomer? Data from the *Arabidopsis* chloroplast proteome has shown that Cpn20 levels are roughly an order of magnitude greater than those of Cpn10(2) [18], [29]. This discrepancy is even greater for the Cpn10(1) homolog, which was not detected in studies of the *A. thaliana* proteome. These levels suggest that the dominant arrangement *in vivo* most likely consists of three Cpn20 molecules with one Cpn10(2) subunit, or one Cpn10(1) subunit. Figure 8 presents a model for the various equilibria that may exist in solution among *A. thaliana* chloroplast co-chaperonins. Our results, together with proteomic data, suggest that hetero-oligomeric co-chaperonins are present in chloroplast in addition to homo-oligomeric Cpn20.

The ability of a small percentage of unique chaperonin (Cpn60) subunits to confer specificity of the system for a distinct client protein was clearly demonstrated by Peng et al. [30], for the Cpn60β4 subunit, which is incorporated in small amounts into the oligomer and dictates specificity of the chaperonin for folding of the NdhH protein. Deletion of Cpn60β4 did not affect housekeeping functions of the plant compared to wild type, although a specific function of the chaperonin complex was impaired. For the co-chaperonins, Cpn10(1) and Cpn10(2) can be found at concentrations much lower than those of Cpn20, and knock-out strains of these genes in *A. thaliana* exhibit a wild-type phenotype under the tested conditions [24]. In a similar manner to the Cpn60β4, it seems likely that their incorporation into co-chaperonin hetero-oligomers even at a low ratio is likely to affect the biogenesis of specific proteins that have yet to be discovered.

A sequence alignment of the three *Arabidopsis* co-chaperonins [17] shows that, unlike the Cpn10-like domains of Cpn20, Cpn10(1) and Cpn10(2) both lack a roof loop. Furthermore, Cpn20, Cpn10(1) and Cpn10(2) vary slightly in the sequences of their mobile loops. These differences may affect the affinity of the co-chaperonin to the chaperonin and influence the size of the formed cavity for substrate folding. A combination of the different properties of each co-chaperonin homolog with the various possible oligomeric arrangements may allow for greater flexibility of protein refolding in chloroplasts. We hypothesize that the ability to adapt to specific substrates can be modulated by changes in the Cpn10/Cpn20 composition.
